# Effect of alirocumab on coronary plaque in patients with coronary artery disease assessed by optical coherence tomography

**DOI:** 10.1186/s12944-021-01528-3

**Published:** 2021-09-12

**Authors:** Fei Gao, Zhi Jian Wang, Xiao Teng Ma, Hua Shen, Li Xia Yang, Yu Jie Zhou

**Affiliations:** Department of Cardiology, An Zhen Hospital, Capital Medical University, Anzhenli avenue, Chao Yang district, Beijing, 100029 China

**Keywords:** Coronary artery disease, PCSK9 inhibitors, Statins, Coronary plaque, Optical coherence tomography

## Abstract

**Background:**

Proprotein convertase subtilisin kexin type 9 (PCSK9) inhibitors have been demonstrated to produce significantly greater reduction in LDL cholesterol levels and cardiovascular events than standard statin therapy. However, evidence on the impact of PCSK9 inhibitors on coronary plaque composition and morphology is limited.

**Methods:**

In this open-label randomized study, eligible patients with intermediate coronary lesions and elevated LDL cholesterol values were randomized to either alirocumab 75 mg Q2W plus statin (atorvastatin 20 mg/day or rosuvastatin 10 mg/day) therapy or standard care. Optical coherence tomography (OCT) assessments for target lesions were obtained at baseline and at 36 weeks of follow-up.

**Results:**

LDL cholesterol levels were significantly decreased in both the alirocumab and standard care arms, whereas the absolute reduction in LDL cholesterol was significantly greater in patients treated with alirocumab (1.72 ± 0.51 vs. 0.96 ± 0.59, *P* < 0.0001). Compared with standard care, the addition of alirocumab to statins was associated with significantly greater increases in minimum fibrous cap thickness (18.0 [10.8–29.2] μm vs 13.2 [7.4–18.6] μm; *P* = 0.029), greater increases in minimum lumen area (0.20[0.10–0.33] mm^2^ vs 0.13 [0.12–0.24] mm^2^; *P* = 0.006) and a greater diminution in maximum lipid arc (15.1̊ [7.8–24.5] vs. 8.4̊ [2.0–10.5]; *P* = 0.008).

**Conclusions:**

The addition of alirocumab to statins can not only provide additional LDL cholesterol lowering effects but also have a potential role in promoting a more stable plaque phenotype.

**Trial registration:**

ClinicalTrials.gov Identifier: NCT04851769. Registered 2 Mar 2019.

## Introduction

Low-density lipoprotein (LDL) cholesterol lowering therapy with statins is the cornerstone for effective treatment of coronary artery disease [1, 2]. However, a substantial proportion of patients cannot achieve target LDL cholesterol levels or tolerate effective doses of statin therapy [3, 4]. In fact, markedly increased residual cardiac risks are observed in this population [1]. Proprotein convertase subtilisin kexin type 9 (PCSK9) inhibitors can reduce intrahepatic degradation of LDL receptors, which leads to increased hepatic expression of LDL receptors and reduced LDL cholesterol levels [4, 5]. Evidence shows that the addition of PCSK9 inhibitors is associated with significantly greater reductions in LDL cholesterol levels and adverse cardiovascular events compared to statins [5–7]. Therefore, current guidelines recommend the use of PCSK9 inhibitors for patients at high cardiovascular risk who cannot reach target LDL cholesterol levels despite maximally tolerated statins [1, 2].

Recently, a series of intravascular ultrasound (IVUS) studies demonstrated that PCSK9 inhibitors in addition to statins achieved significantly greater atheroma volume regression than statin monotherapy [8–11]. However, due to the limited spatial resolution, IVUS is unable to evaluate the effects of PCSK9 inhibitors on fibrous cap thickness (FCT), which has been confirmed as an important indicator of plaque vulnerability [12, 13]. Although it has been well established that LDL cholesterol lowering therapy with statins has favorable effects on reducing coronary atheroma burden and increasing fibrous cap thickness [14, 15], it remains unclear whether the addition of PCSK9 inhibitors could further improve FCT. Optical coherence tomography (OCT) is currently the gold standard for evaluating the small changes in FCT [16, 17]. Therefore, the aim of this study was to evaluate the effects of PCSK9 inhibitors on fibrous cap thickness by OCT imaging in patients with intermediate coronary lesions.

## Methods

The study is an open-label, single-center, randomized study involving patients with intermediate coronary lesions (50–70% diameter stenosis). From March 2019 to Jan 2020, all consecutive patients who received coronary angiogram and OCT imaging in An Zhen Hospital (Beijing, China), which is a teaching hospital in which over 15,000 percutaneous coronary intervention procedures are performed each year, were evaluated. Eligible patients included those who were (I) 18–80 years of age, (II) diagnosed with stable coronary artery disease or acute coronary syndrome (ACS) on admission, (III) planned to have clinically indicated coronary angiography and identified wit’h at least one intermediate lesion (50–70% diameter stenosis) on de novo coronary arteries, (IV) identified with elevated LDL cholesterol values (LDL cholesterol ≥1.81 mmol/L [≥70 mg/dL] for patients with ACS, or ≥ 2.59 mmol/L [≥100 mg/dL] for non-ACS patients) despite taking rosuvastatin 10 mg/day or atorvastatin 20 mg/day for 2–4 weeks or with maximally tolerated statin therapy, and (V) able to provide written, informed consent. The study inclusion and exclusion criteria are listed in Table 1.

**Table 1:** Inclusion and Exclusion criteria

Inclusion criteria	Exclusion criteria
18–80 years of age	Known hypersensitivity or contraindications to alirocumab and/or statin therapy
Diagnosed as stable coronary artery disease or acute coronary syndrome	Received balloon angioplasty or stent implantation for target lesion
Received OCT imaging measurement	Unable to conduct OCT imaging analysis
LDL cholesterol values ≥1.81 mmol/L for patients with ACS or ≥ 2.59 mmol/L for non-ACS patients despite statin therapy	Prior usage of PCSK9 inhibitors
At least one intermediate lesion (50–70% diameter stenosis) in de novo coronary arteries	Severe renal dysfunction (creatinine clearance < 30 mL/min)
Provided written informed consent	Severe hepatic dysfunction
	Baseline triglyceride > 400 mg/dl
	History of hemorrhagic stroke
	Pregnant or breast-feeding women
	Life expectancy < 1 year
	Inappropriate for the study for any reason based on the investigators’ judgement

*LDL* Low-density lipoprotein, *PCSK9* proprotein convertase subtilisin kexin type 9, *OCT* optical coherence tomography, *ACS* acute coronary syndrome

**Fig. 1:**
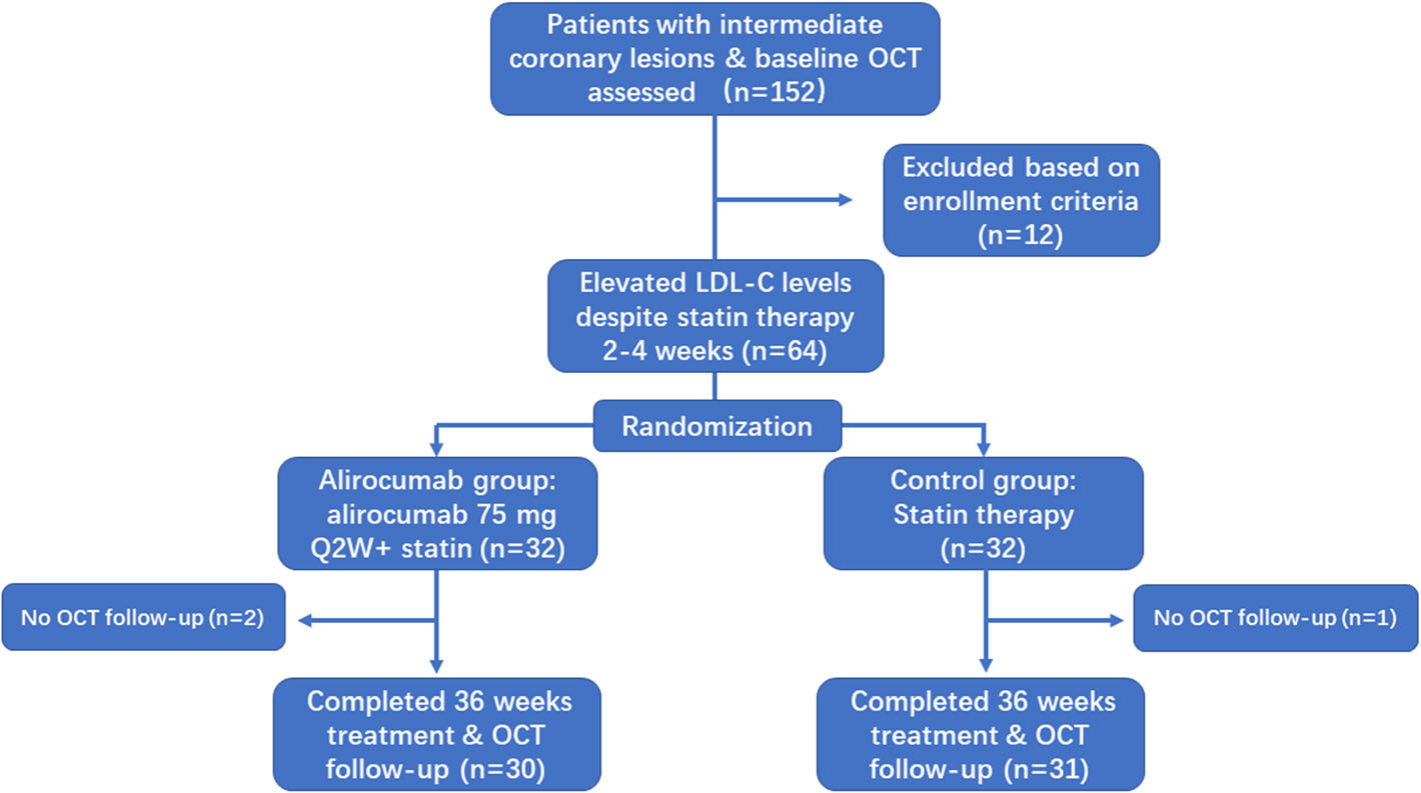
Study flow

The study included a 36-week open-label treatment period (including posttreatment OCT imaging), initiating within 4 weeks of baseline coronary angiogram (Fig. 1). During the open-label treatment period, patients were randomized 1:1 to either the alirocumab arm or the standard care arm. Patients in the alirocumab arm received alirocumab 75 mg Q2W on top of standard statin therapy (atorvastatin 20 mg/day or rosuvastatin 10 mg/day). The last dose of alirocumab was given at week 34. Patients in the standard care arm continued to receive atorvastatin 20 mg/day or rosuvastatin 10 mg/day. Statin dose escalation or the addition of other concomitant nonstatin lipid-lowering therapies could be considered by the physicians responsible for achieving the target LDL cholesterol levels. Antithrombotic therapy and other concomitant medications were exclusively decided by their responsible physicians. Follow-up coronary angiograms and OCT imaging analyses of the same vessels were carried out at the end of the treatment period in both arms (at week 36 ± 2 weeks). Regular medical examinations and laboratory tests were conducted at 4, 12 and 36 weeks. All enrolled patients were monitored and evaluated for medical adherence to lipid lowering therapy, safety and any other adverse events during the study period. LDL cholesterol, HDL cholesterol, and triglycerides were measured by the central laboratory. LDL cholesterol was calculated according to the Friedewald formula [18]. The study was approved by the local medical ethics committee.

### Optical coherence tomography

OCT images were obtained at baseline and at the 36 ± 2-week follow-up. Optical coherence tomography imaging systems (Optis or Ilumien Imaging System™, Abbott Vascular, USA) were used. Testing on contrast injection was required to ensure a complete wash-out of the vessel. The entire length of the target lesions (including 5 mm proximal and distal segments) was evaluated. All imaging analyses were performed by independent investigators who were blinded to the study protocol. Target lesions between baseline and follow-up OCT were matched and compared based on the distance from the landmarks (e.g., calcifications and branches). Calibration was required before image analysis. Values of minimum lumen area, minimum FCT, and maximum lipid arc were measured. The target plaque was characterized using previously established criteria [19].

### Study endpoints

The primary endpoint of the study was the OCT derived absolute changes in minimum fibrous cap thickness between baseline and follow-up. Secondary endpoints included the absolute changes in minimum lumen area between baseline and follow-up, as well as the absolute changes in maximum lipid arc. Minimum fibrous cap thickness was defined as the smallest FCT in the three candidate frames selected by manual screening. The maximum lipid arc was defined as the largest lipid arc from the center of the lumen. The minimum lumen area was defined as the smallest lumen area in the target lesion calculated by automated measurements and manual corrections.

In addition, the incidence rates of major adverse cardiac events (defined as the composite outcome of death, myocardial infarction, and ischemia-driven target lesion revascularization) and treatment related adverse reactions during the follow-up period were recorded. Myocardial infarction was defined according to the fourth universal definition of myocardial infarction [20]. Ischemia-driven target lesion revascularization was defined as reintervention driven by anginal symptoms and significant angiographic stenosis (> 70% diameter stenosis).

### Statistical analysis

Continuous variables are reported as the means ± standard deviations or median (interquartile ranges). Categorical variables are presented as counts and percentages. Continuous variables between the two groups were compared by the Mann–Whitney U test. Comparisons of continuous variables between baseline and follow-up were performed by the Wilcoxon signed rank test. Comparisons of categorical variables were performed by the Fisher’s exact test. A two-tailed test *P* value < 0.05 was considered as statistically significant. The SPSS Statistics 25.0 package was used.

## Results

A total of 61 eligible patients (31 patients in the standard care arm and 30 patients in the alirocumab arm) with complete clinical and OCT imaging follow-up were analyzed (Fig. 1). Nearly half of the patients (15/31) in the standard care arm received ezetimibe and statin combination therapy. The baseline and procedural characteristics of the two groups are listed in Table 2. Of note, patients were predominantly male and had a high percentage of guideline recommended medical therapies in both groups. All the participants were prescribed antiplatelet therapy, and approximately 90% of them were treated with beta blockers.

**Table 2:** Baseline characteristics

	Standard of care (***N*** = 31)	Alirocumab (***N*** = 30)
Age, yrs	61.3 ± 9.9	61.3 ± 8.9
Male	74.2 (23)	66.7 (20)
Diabetes	25.8 (8)	23.3 (7)
Current smoker	25.8 (8)	30 (9)
Hypertension	61.3 (19)	56.7 (17)
Prior MI	9.7 (3)	13.3 (4)
Prior stroke	3.2 (1)	10.0 (3)
ACS	41.9 (13)	36.7 (11)
Antiplatelet	100 (31)	100 (30)
Beta-blocker	90.3 (28)	93.3 (28)
ACEI/ARB	64.5 (20)	60.0 (18)
Chronic statin before enrollment	32.3 (10)	26.7 (8)
Imaged artery		
Left anterior desending	41.9 (13)	43.3 (13)
Left circumflex	22.6 (7)	26.7 (8)
Right coronary	32.2 (10)	30.0 (9)
Others	3.2 (1)	0

*ACS* acute coronary syndrome, *MI* myocardial infarction, *ACEI/ARB* angiotensin-converting enzyme inhibitors/angiotensin receptor blockers

Biochemical measures throughout the study are summarized in Table 3. At baseline, no significant differences were observed between the standard care arm and the alirocumab arm. At week 36, LDL cholesterol levels were significantly decreased in both groups compared with baseline, from 3.18 mmol/L to 2.22 mmol/L (*P* < 0.0001) in the standard care arm and from 3.04 mmol/L to 1.32 mmol/L in the alirocumab arm (*P* < 0.0001). However, the absolute changes in LDL cholesterol levels were significantly higher in patients treated with alirocumab (1.72 ± 0.51 vs. 0.96 ± 0.59, *P* < 0.0001). In addition, patients in alirocumab arm demonstrated favorable changes in triglyceride levels, but the differences were not significant. C-reactive protein (CRP) levels decreased in both groups, but no significant difference was observed between the groups.

**Table 3:** Biochemical parameters

	Standard of care (***N*** = 31)	Alirocumab (***N*** = 30)	***P*** Value
LDL cholesterol, mmol/L			
Baseline	3.18 ± 0.97	3.04 ± 0.78	–
After 36 weeks treatment	2.22 ± 0.69	1.32 ± 0.39	< 0.0001
Changes from baseline	−0.96 ± 0.59	−1.72 ± 0.51	< 0.0001
HDL cholesterol, mmol/L			
Baseline	1.30 ± 0.41	1.41 ± 0.61	–
After 36 weeks treatment	1.38 ± 0.43	1.48 ± 0.47	0.35
Changes from baseline	0.08 ± 0.36	0.07 ± 0.38	0.74
Triglycerides, mmol/L			
Baseline	1.56 (1.19 to 2.38)	1.84 (1.19 to 2.56)	–
After 36 weeks treatment	1.53 (1.09 to 2.26)	1.54 (1.00 to 2.09)	0.68
Changes from baseline	−0.05 (0.64 to 1.42)	−0.29 (− 0.96 to 0.35)	0.077
CRP, mg/L			
Baseline	1.62 (0.90 to 3.00)	1.69 (0.75 to 3.37)	–
After 36 weeks treatment	1.10 (0.89 to 2.50)	1.59 (0.92 to 2.61)	0.64
Changes from baseline	0.54 (−0.46 to 1.34)	0.12 (−0.74 to 1.08)	0.50

*CRP* C-reactive protein, *LDL* low-density lipoprotein, *HDL* high-density lipoprotein

OCT-derived baseline and follow-up parameters are listed in Table 4. Significantly greater increases in the changes in minimum FCT (18.0 [10.8–29.2] μm vs. 13.2 [7.4–18.6]μm; *P* = 0.029) and the changes of minimum lumen area (0.20[0.10–0.33]mm^2^ vs. 0.13 [0.12–0.24]mm^2^; *P* = 0.006) were observed in the alirocumab group compared to the standard care group. Similarly, the absolute changes in the maximum lipid arc were also significantly greater in the alirocumab group than in the standard care group (15.1̊ [7.8–24.5] vs. 8.4̊ [2.0–10.5]; *P* = 0.008). Additionally, patients in the alirocumab arm demonstrated a trend of greater but not statistically significant reduction in the percentage of thin-cap fibroatheroma (TCFA) compared to those in the standard care arm (3.3% vs. 16.1%; *P* = 0.09).

**Table 4:** OCT -derived study endpoints

	Standard of care (***N*** = 31)	Alirocumab (***N*** = 30)	***P*** Value
Minimum fibrous cap thickness, um			
Baseline	116.4 (90.1 to 136.2)	126.0 (87.5 to 145.5)	0.44
After 36 weeks treatment	124.2 (98.2 to 144.3)	144.0 (111.5 to 151.8)	0.049
Changes from baseline	13.2 (7.4 to 18.6)	18.0 (10.8 to 29.2)	0.029
Maximum lipid arc, degree			
Baseline	110.9 (90.2 to 132.4)	109.6 (89.8 to 130.0)	0.53
After 36 weeks treatment	102.2 (87.0 to 123.1)	93.5 (77.5 to 108.1)	0.19
Changes from baseline	−8.4 (−2.0 to −10.5)	−15.1 (−7.8 to − 24.5)	0.008
Minimum lumen area, mm^2^			
Baseline	2.47 (2.20 to 2.74)	2.32 (2.07 to 2.63)	0.22
After 36 weeks treatment	2.60 (2.19 to 2.90)	2.57 (2.27 to 2.90)	0.77
Changes from baseline	0.13 (0.12 to 0.24)	0.20 (0.10 to 0.33)	0.006
TCFA, %(n)			
Baseline	25.8 (8)	20.0 (6)	0.59
After 36 weeks treatment	16.1 (5)	3.3 (1)	0.09

*TCFA* thin-cap fibroatheroma

No death or myocardial infarction event was recorded in either the alirocumab or standard care arm (Table 5). However, 1 patient suffered from ischemia driven target lesion revascularization in the standard care arm, but not in the alirocumab arm. The incidence of treatment-related adverse reactions was similar in both groups. Nasopharyngitis (standard care: 1 patient; alirocumab: 2 patients) was the most common reaction. Two (6.7%) patients in the alirocumab group experienced local injection-site reactions. All the treatment-related adverse reactions found in this study were recorded as mild in intensity, and all the participants were tolerated following their treatment plan.

**Table 5:** Clinical events

	Standard of care (*N* = 31)	Alirocumab (*N* = 30)
Adverse cardiac events	0	0
Cardiac death	0	0
Myocardial infarction	1	0
Ischemia driven target lesion revascularization,		
Treatment-related adverse events	1	2
Nasopharyngitis	1	2
Injection-site reaction	0	1
Back pain	1	1
Transaminase elevation		

## Discussion

The results of our study indicated that the addition of alirocumab was associated with a significantly greater reduction in LDL cholesterol levels, a greater increase in FCT, and a greater diminution in maximum lipid arc, compared with standard lipid lowering therapy.

The impact of lipid lowering therapy on atheroma plaque morphology was initially established in statin trials [21]. Coronary plaque regression can be achieved when the decrease in LDL cholesterol levels exceeds 50% due to the treatment of statins [22]. However, our previous epidemiological study indicated that large numbers of patients cannot achieve sufficient LDL cholesterol reductions despite treatment with statin therapy [3]. PCSK9 inhibitors are novel pharmacologic agents, and it has been shown that the addition of PCSK9 inhibitors to statins can further reduce LDL cholesterol levels by 43 to 64% [23]. However, evidence regarding the effect of PCSK9 inhibitors on atheroma plaque stability is limited. The ODYSSEY J-IVUS trials showed that the addition of alirocumab to statins resulted in a numerical greater reduction in total atheroma volume, but it did not attain statistical significance due to the limited sample size [10]. Conversely, the GLAGOV trial reported a significant reduction in atheroma volume in patients treated with evolocumab for 76 weeks [8]. However, the GLAGOV trial showed that the addition of evolocumab did not produce differential changes in plaque composition by IVUS assessment compared to statin monotherapy. Although the addition of PCSK9 inhibitors was shown to reduce atheroma volume in these two studies, there is a gap of evidence regarding the impact of PCSK9 inhibition on other vulnerable plaque features, such as fibrous cap thickness. OCT imaging analysis, which has 10 times greater resolution than IVUS, is an ideal imaging modality to assess fibrous cap thickness of coronary plaque [17, 24]. A recent retrospective, observational OCT study revealed that evolocumab provided a greater increase in FCT in patients with recent acute coronary syndrome than statin monotherapy [25]. To date, there is only one randomized study evaluating the effects of PCSK9 inhibitors on plaque morphology by OCT – the ALTAIR study [26]. It enrolled 24 patients randomized to either alirocumab or statins (rosuvastatin 10 mg/d). It also showed a significantly greater increase in fibrous cap thickness in the alirocumab group than in the statin group. However, in the current study nearly half of the patients in the standard care arm received ezetimibe and statin combination therapy. It was close to real-world clinical practice since the recent guidelines also recommended adding an ezetimibe before initiating PCSK9 inhibitors for patients with elevated LDL cholesterol levels. The results of this study were generally consistent with previous studies and demonstrated a significantly greater increase in minimum lumen area in the alirocumab arm assessed by OCT imaging. In addition, the alirocumab arm was associated with a significantly greater increase in FCT and a greater reduction in maximum lipid arc compared with the standard care arm. The current results indicated that the addition of alirocumab can not only provide profound LDL cholesterol lowering effects but also have a potential role in improving the stability of the plaque.

**Fig. 2:**
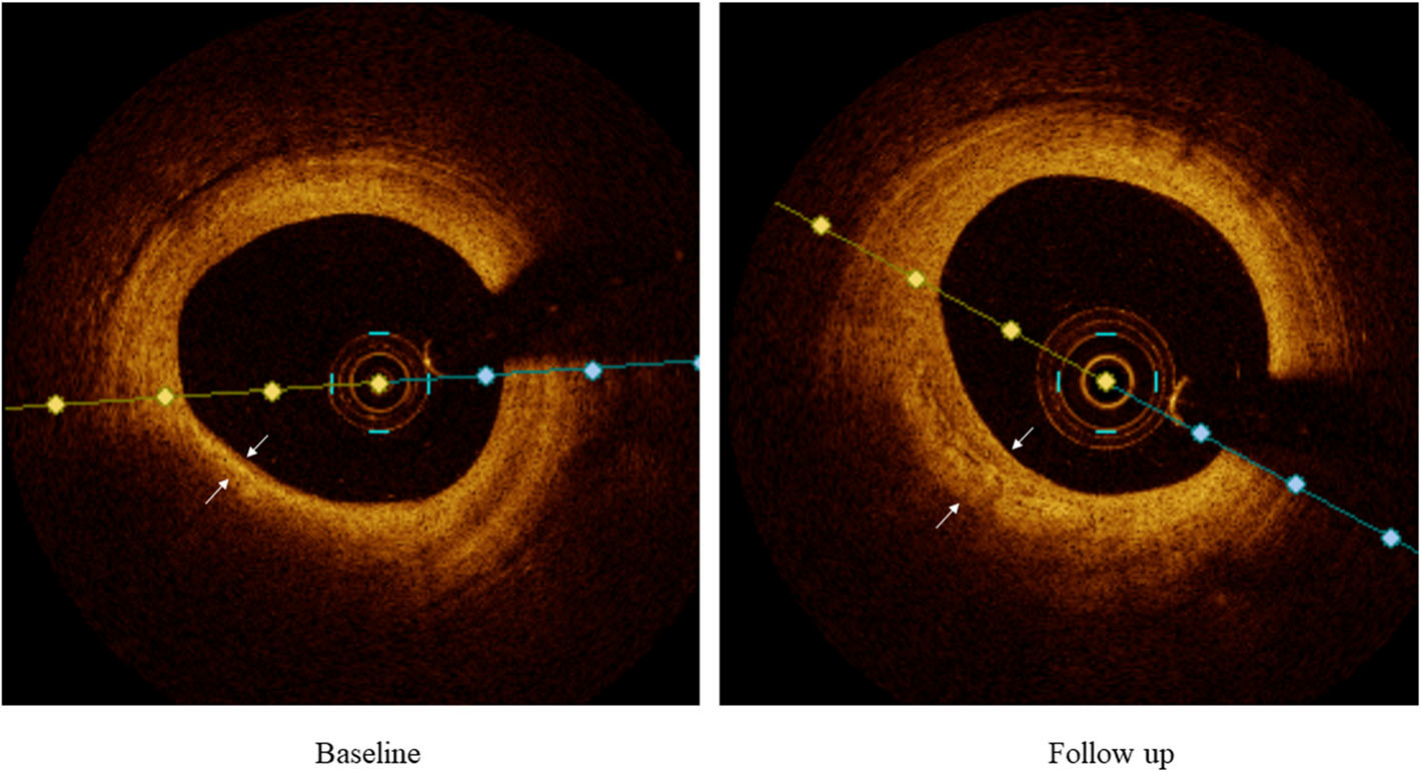
Representative OCT imaging. Changes in fibrous cap thickness (white arrows) between baseline and follow-up in a patient with alirocumab

Although statins have demonstrated a significant CRP lowering effect, and the degree of CRP lowering was correlated with the extent of plaque regression [27, 28] and cardiovascular event risk reduction [29], it remains controversial whether PCSK9 inhibitors have similar effects on CRP levels. A recent Mendelian randomization study found strong associations of decreased PCSK9 concentrations and increased CRP levels [30]. In this study, no incremental reductions in CRP levels were observed in patients treated with alirocumab. However, the study might be underpowered to detect the differences in CRP between the groups; thus, the differences need to be further evaluated in large studies. However, the study results suggested that the favorable effects of alirocumab on the plaque phenotype were independent of CRP changes. A previous meta-analysis also indicated that the reduction in coronary atherosclerosis observed with nonstatin lipid-lowering therapy was attributed to the degree of LDL cholesterol lowering [31]. Therefore, it seems reasonable to believe that the effects of PCSK9 on coronary plaque might be mediated by LDL cholesterol lowering Fig. 2.

### Study strength and limitations

The current trial is the first randomized OCT imaging trial to show the superiority of alirocumab over standard lipid lowering therapy (including high-intensity statin and ezetimibe combination therapy) on plaque modification in patients with intermediate coronary lesions. However, there are several potential limitations to this study. The first major limitation is the relatively short treatment duration; therefore, the long-term effects of PCSK9 inhibitors on plaque, as well as their clinical prognosis cannot be evaluated. The second limitation is the open nature of this study. The person who conducted the OCT imaging scans and analyses were unaware of the patients’ allocation. However, the patients and treating physicians in this study were unblinded, which might influence the treatment benefits. The third limitation is that the study did not record and compare medical adherence except for lipid lowering agents. Since recent evidence showed that PCSK9 inhibitors could positively affect treatment adherence and patients’ quality of life [32, 33], it may introduce potential bias into the study. Another important limitation is that there is no compelling evidence demonstrating a direct link between the increase in OCT-derived fibrous-cap thickness and the improvement in cardiovascular prognosis. However, a series of pathological studies certified that FCT is a major determinant of vulnerable coronary plaque [12, 34]. Furthermore, it is well acknowledged that OCT-defined thin-cap fibroatheroma is correlated with vulnerable plaque characters identified by other imaging techniques [35], all of which have a strong link to future adverse clinical events [23, 36]. Therefore, to date, the increase in fibrous cap thickness has been considered a representation of coronary plaque stabilization [37–40].

## Conclusions

Among patients who cannot achieve LDL cholesterol targets despite standard lipid lowering therapy, the addition of alirocumab can not only provide a greater reduction in LDL cholesterol, but also have a favorable effect on improving the vulnerability of the plaque. Further studies are needed to clarify the clinical implications of these results measured by OCT.

## Data Availability

The datasets used and/or analyzed during the current study are available from the corresponding author on reasonable request.

## Abbreviations

LDL: Low-density lipoprotein
PCSK9: Proprotein convertase subtilisin kexin type 9
IVUS: Intravascular ultrasound
OCT: Optical coherence tomography
FCT: Fibrous cap thickness
ACS: Acute coronary syndrome
CRP: C-reactive protein
MI: Myocardial infarction
ACEI/ARB: Angiotensin-converting enzyme inhibitors/angiotensin receptor blockers
TCFA: Thin-cap fibroatheroma
